# Vitamin B12 deficiency in a young male with Imerslund-Gräsbeck syndrome: case report

**DOI:** 10.3389/fped.2025.1645600

**Published:** 2025-09-02

**Authors:** Lina Xing, Yujie Guo, Yang Li, Xuquan Zhou, Fengru Lin, Yan Wang

**Affiliations:** Department of Hematology, Hebei Key Laboratory of Hematology, The Second Hospital of Hebei Medical University, Shijiazhuang, China

**Keywords:** vitamin b12 deficiency, Imerslund-Gräsbeck syndrome, AMN gene, megaloblastic anemia, proteinuria

## Abstract

Imerslund-Gräsbeck syndrome (IGS) is a rare genetic disorder characterized by selective vitamin B12 deficiency co-existing with asymptomatic proteinuria. It is caused by bi-allelic mutations in either the *CUBN* or *AMN* gene, which encode the two protein components of the cobalamin-intrinsic factor receptor. Patients stay healthy with lifelong parenteral administration of vitamin B12. Here, we report a case of a young male who presented with severe macrocytic anemia and asymptomatic proteinuria from the age of one year. His low serum level of vitamin B12 suggested vitamin B12 deficiency. Further, the patient was heterozygous for the *AMN* variant c.1006 + 34_1007-31 del mutation with duplication of exons 2–3, indicating a definite diagnosis of typical IGS. He was treated by administration of vitamin B12 injections, resulting in rapid improvement of hemoglobin levels. However, the previously detected proteinuria was found to persist at follow-up.

## 1 Introduction

Imerslund-Gräsbeck syndrome is a rare genetic syndrome characterized by selective vitamin B12 deficiency concomitant with asymptomatic proteinuria (1). It has varying manifestations, including megaloblastic anemia, neurological signs, gastrointestinal or respiratory infections, and other rare symptoms due to low levels of cobalamin (2). As the deficiency of the cobalamin-intrinsic factor receptord affects the absorption of vitamin B12 from the ileum into the blood, patients respond well to parenteral vitamin B12 therapy, but not to oral vitamin B12 supplementation (3, 4). To date, more than four hundred cases of this condition have been published and reported (5). In this article, we report a case of a young male who presented with macrocytic anemia and proteinuria since early childhood, and was finally diagnosed with Imerslund-Gräsbeck syndrome by mutational analysis.

## 2 Case presentation

A 22-year-old young man was admitted to our center owing to complaints of fatigue and pallor for more than 21 years. These symptoms had worsened six months ago. Atthe age of 10 months old, the patient's parents noticed that the patient was pallor without failure to thrive. The patient presented to the hosipital found severe macrocytic anemia (hemoglobin 60 g/L) with low serum vitamin B12 level and mild proteinuria (proteinuria of 1+).Hence, the diagnosis of megaloblastic anemia was confirmed, which were ameliorated to an extent with red blood cell transfusion and vitamin B12 injections. Since then, vitamin B12 was supplemented when the patient felt fatigue or pallor, but intramuscular injection of vitamin B12 was more effective than oral administration. No family history of relevance and no history of dietary restrictions. The physical examination showed no positive signs other than pallor, without failure to thrive and intellectual impairment at this present time.

**Figure 1:**
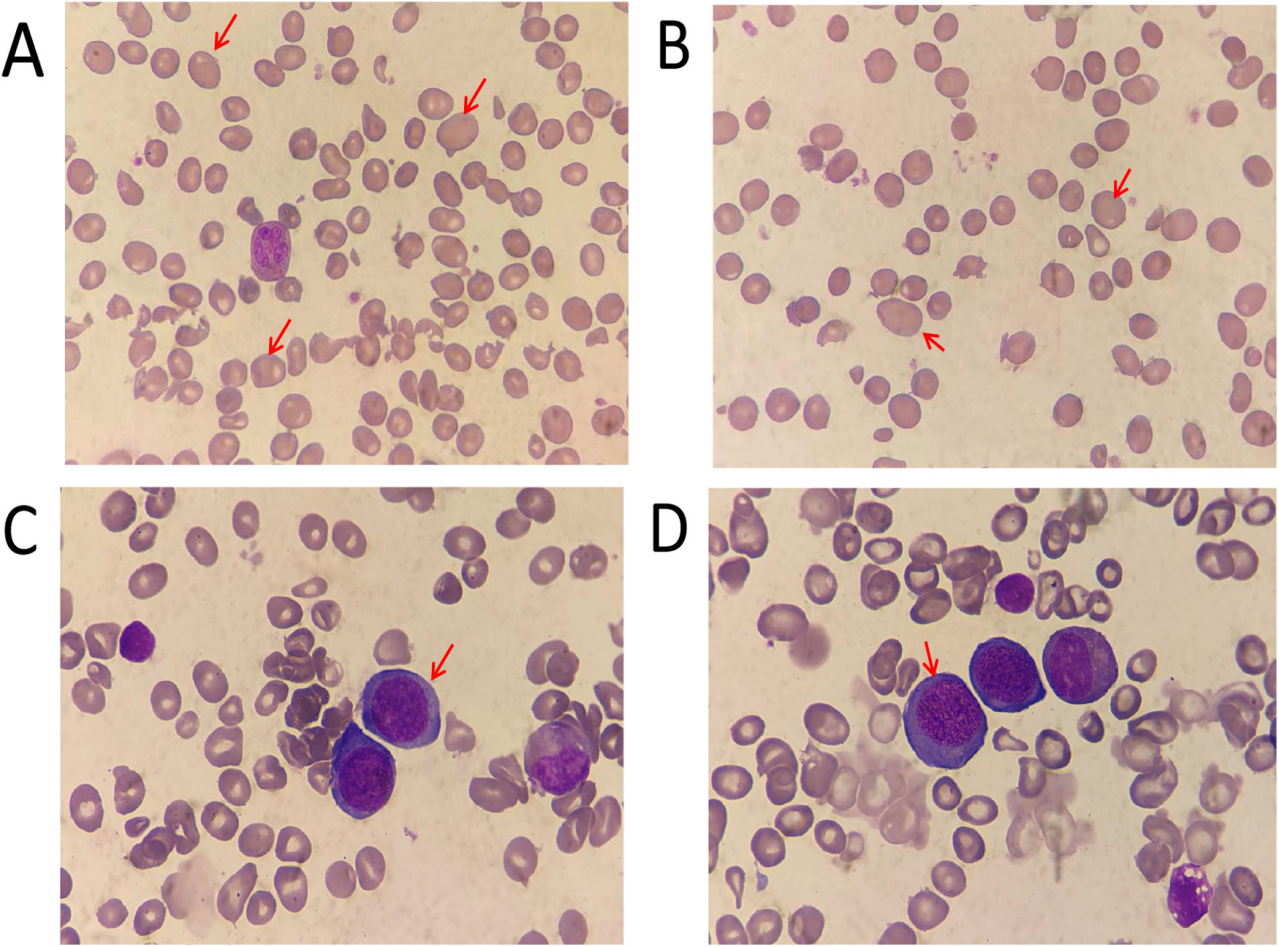
Morphology analysis (giemsa staining)of the peripheral blood **(A,B)** ×1,000 and bone marrow **(C,D)** ×1,000 aspirate. The red arrows indicate megaloblastic mature red blood cells and mesenchymal red blood cells.

The clinical features are summarized in Table 1. These parameters of hemogram were indicative of macrocytic anemia. Urine investigations further showed mild proteinuria with normal renal function tests. Ultrasonogram of the abdomen was normal.Antibodies against gastric parietal cells and intrinsic factor were negative. Megaloblastic change was observed in peripheral blood and bone marrow smear analysis with hyperplasia (Figure 1), and the karyotype was 46,XY [20].

**Table 1:** Clinical features of the patient.

Clinical features	Results	Normal range
Age at onset, months	10	
Age at diagnosis, years	22	
Gender	Male	
White blood cell, ×10^9^/L	3.53	3.5–9.5
Hemoglobin, g/L	56	125–160
Mean corpuscular volume, fl	112.8	82–100
Reticulocyte ratio, %	3.31	0.5–1.5
Platelets, ^9^/L	180 × 10	125–350
Serum vitamin B12, pg/ml	84	211–911
Serum folate, ng/L	15.8	＞3.38
Serum ferritin, ng/ml	235.4	10–291
Coombs test	Negative	Negative
Antibodies against intrinsic factor	Negative	Negative(
Antibodies against gastric parietal cells	Negative	Negative
Proteinuria	1+	Negative
Urinary protein, g/24 h	0.88	0–0.15
Serum creatinine, µmol/L	67	41–73
eGFR, ml/(min*1.73 m^2^)	119.36	
Bone marrow smear	Erythromegaloblastosis	
Karyotype	46,XY [20]	46,XY
*AMN* gene mutation		
Allele 1	c.1006 + 34_1007-31 del	
Allele 2	Duplication of exons 2–3	

**Figure 2:**
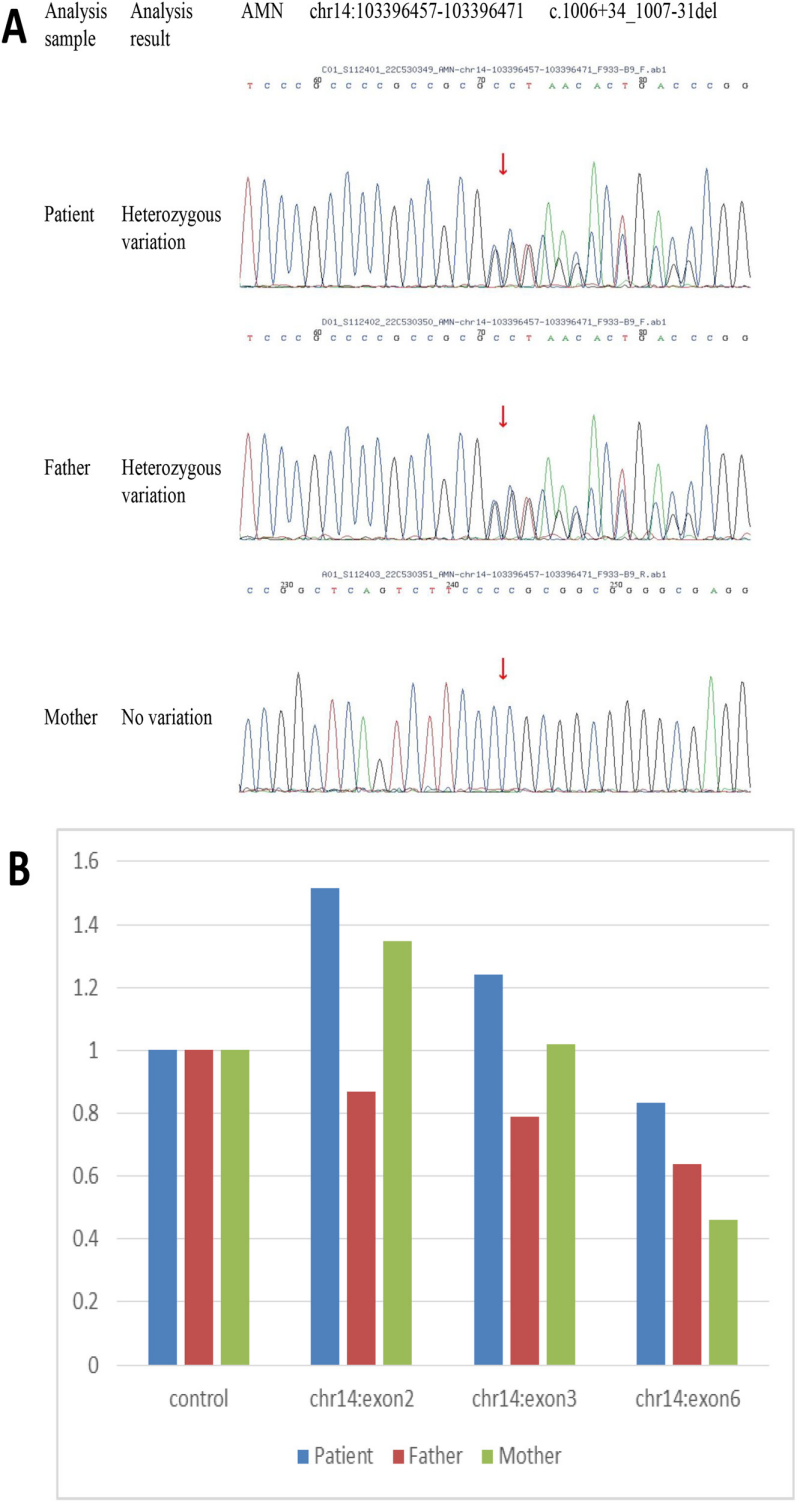
DNA sequencing results for peripheral blood samples from the patient and his parents. Heterozygosity variant c.1006 + 34_1007-31 del of *AMN* was detected in the patient and his father through sequencing **(A)** Sequence analysis showed the duplication of exons 2–3 of *AMN* acquired from the patient's mother **(B)**.

The clinical and laboratory results indicated macrocytic anemia and slight proteinuria with a severe lack of vitamin B12. On this basis, the patient was diagnosed with Imerslund-Gräsbeck syndrome (IGS). Whole-exome sequencing of DNA from the patient's peripheral blood was conducted at the age of 22, and variants of the exon regions and of the intron region of the *AMN* gene were performed. Sequence analysis revealed the duplication of exons 2–3 of the AMN, i.e., compound heterozygosity with the known pathogenetic intronic variant c.1006 + 34_1007-31 del. Furthermore, the presence of variant c.1006 + 34_1007-31 del was also detected in his father through Sanger sequencing (Figure 2A). The duplication of exons 2–3 of the *AMN* gene had been acquired from his mother (Figure 2B).

**Figure 3:**
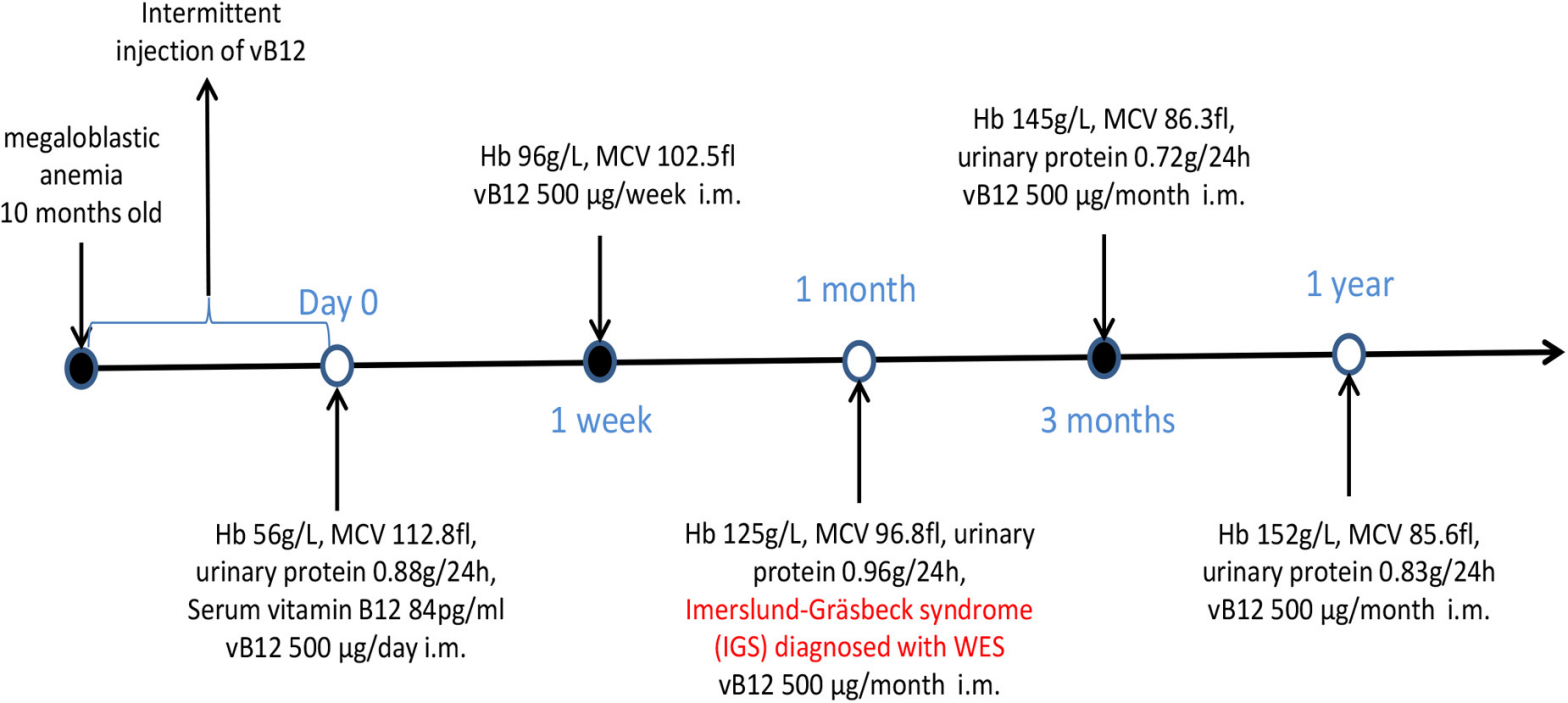
Timeline of case report.

Parenteral vitamin B12 therapy was initiated (500 μg/day i.m. for 7 days), which resulted in rapid improvement of hemoglobin (96 g/L) levels with amelioration of fatigue and pallor. This regimen was followed by parenteral administration of vitamin B12 at a dose of 500 μg weekly for four weeks, and then 500 μg monthly. At follow-up, hemoglobin levels were found to have returned to normal, although proteinuria persisted (Figure 3).

## 3 Discussion

In patients who develop vitamin B12 (cobalamin) deficiency in early childhood, genetic factors should be considered after excluding insufficient dietary intake as a cause. Imerslund-Gräsbeck syndrome (IGS) is a rare autosomal recessive disorder, whose most common manifestation is megaloblastic anemia with mild proteinuria and normal renal functions. Parenteral vitamin B12 therapy is the most effective treatment (1). This syndrome was first described in Finland and Norway, with an estimated prevalence of 1:200,000 among children from a few months to about fourteen years old (6, 7). With growing understanding of the disease, new cases have been reported in worldwide in recent years; however, many patients may still be misdiagnosed.

Patients usually present with non-specific anemic manifestations such as pallor, fatigue, and decreased activity tolerance from an early age. Detailed examination reveals megaloblastic anemia, decreased serum vitamin B12 levels, and Mild proteinuria is observed in more than half of patients with IGS (7–9).Neurological complications, protracted infections, failure to grow and thrive, and amblyopia may manifest as other possible clinical signs of cobalamin deficiency (10).

IGS is caused by a defect in a receptor of the cobalamin-intrinsic factor in the distal small intestine (ileum) (11). The subunits of cubilin (CUBN) and amnionless (AMN) are considered essential components of the receptor for intestinal cobalamin uptake. Two different genes encoding these two protein components has been identified, the *CUBN* gene and *AMN* gene map loci are 10p12.1 (12) and 14q32 (13), respectively. Biallelic mutations of this two genes affecting either cubilin or amnionless are responsible for selective malabsorption of vitamin B12 in men. So the diagnosis of IGS may be accurately made through mutational analysis of the appropriate genes *CUBN* and *AMN.* Because of the specifics of the pathogenesis of IGS, lifelong monthly treatment with intramuscular injection vitamin B12 is required to achieve complete recovery and restoration of hematological parameters to normal (1). The receptor proteins cubilin and amnionless also express in the renal proximal tubules, and they exhibit an interdependent relationship in their role in the reabsorption of filtered low-molecular-weight proteins (14, 15). Mutations in either CUBN or AMN disrupt this reabsorptive process, leading to mild proteinuria, which is a hallmark of IGS (16, 17). The persistence of proteinuria despite vitamin B12 treatment further supports its tubular origin, as the defective receptor function in the kidney is independent of cobalamin metabolism (18).

Our patient presented with macrocytic anemia from early childhood. His main symptoms were associated with anemia, without neurological damage or failure to thrive. Based on the low level of serum vitamin B12 coexisting with unexplained proteinuria, the diagnosis of IGS was favored. In clinical practice, good response to treatment with parenteral vitamin B12 has been observed. Prior to the detection of *AMN* mutations in his family, the already-known pathogenic variant (c.1006-31 del) in the *AMN* gene detected in our patient has been reported (19, 20). Through mutational analysis, the presence of IGS was confirmed and this syndrome was confirmed and lifelong parenteral vitamin B12 therapy was recommended.

## 4 Conclusions

In this case report, we present a 22 year-old male with macrocytic anemia and asymptomatic proteinuria from infancy, the patient was eventually diagnosed with Imerslund-Gräsbeck syndrome through mutational analysis, and showed good response to intramuscular injections of vitamin B12. Imerslund-Gräsbeck syndrome as a rare disorder should be considered within patients excluding insufficient dietary intake, who present with deficiency of Vitamin B12 and mild proteinuria from early childhood, genetic-related examinations are of great significance for precise diagnosis. For people of childbearing age, it is necessary to increase genetic counseling to reduce the incidence of the next generation.

## Data Availability

The datasets presented in this study can be found in online repositories. The names of the repository/repositories and accession number(s) can be found in the article/Supplementary Material.
